# Digital Self-Guided Mental Health Interventions to Prevent Workplace Burnout and Enhance Psychological Wellness: Protocol for a Systematic Review

**DOI:** 10.2196/80417

**Published:** 2026-01-12

**Authors:** Ehsan Etezad, John Fiset, Raghid Al Hajj

**Affiliations:** 1 Applied Organizational Psychology, Applied Science Department, Saint Mary's University, 923 Robie Street, Halifax, B3H 3C3, Canada, 1 902-420-5400; 2 Management Department, Sobey School of Business, Saint Mary's University, Halifax, Canada; 3 Business Administration Department, Gulf University for Science & Technology, Hawally, Kuwait

**Keywords:** burnout, job stress, mental health, psychological well-being, self-guided intervention, workplace wellness

## Abstract

**Background:**

Employee burnout has reached critical levels, with nearly 40% of workers reporting symptoms driven by excessive workloads, inadequate managerial support, and toxic organizational cultures. Research has consistently indicated that workplace stress significantly impacts employees’ physical health. Consequently, a variety of self‑guided mental health programs have been developed to address these challenges. Despite numerous evidence‑based self‑guided mental health interventions, there remains a lack of understanding and clarity regarding which specific content modules, intervention features, active components, and theoretical frameworks most effectively drive meaningful improvements in workplace mental health.

**Objective:**

This systematic review aims to synthesize evidence on self-guided digital mental health interventions in workplace settings. Specifically, it seeks to identify which content, design features, activities, and assignments are most effective for preventing burnout and enhancing psychological well-being. This review will also examine underlying theoretical mechanisms and assess the methodological rigor of included studies to provide actionable recommendations for intervention developers and organizational stakeholders.

**Methods:**

A systematic search will be executed across PsycINFO, PubMed, Web of Science, and Cochrane Library for relevant studies published since 2000, using comprehensive search strings targeting working adults, digital delivery modes, outcomes related to burnout prevention, stress reduction, well-being, and controlled experimental designs. Titles, abstracts, and keywords will be screened, with additional records identified through manual searches of reference lists. Following the removal of duplicates, a 2-step screening process will be applied to studies based on the defined inclusion and exclusion criteria. Data from eligible studies will be extracted into a standardized Excel template covering authors, sample characteristics, study design, intervention content and theoretical framework, outcome measures, intervention effectiveness, implementation fidelity, and risk-of-bias assessment.

**Results:**

Preliminary searches were conducted in early 2025. The review is anticipated to be completed by May 2026.

**Conclusions:**

This review will identify theoretical mechanisms and core components driving the effectiveness of self-guided digital interventions for workplace burnout, stress, and psychological well-being. By identifying theoretical background, rationale, content, activities, characteristics, and implementation factors behind evidence-based digital interventions, the findings will guide the development of scalable and accessible programs that enhance employee wellness, boost productivity, and inform future organizational mental health and workplace wellness initiatives.

## Introduction

### Overview

 In North America, approximately 40% of employees experience burnout, driven primarily by heavy workloads, insufficient managerial support, and toxic work cultures [1,2]. This increased level of burnout has tangible consequences: 7.5% of the employed population has taken time off due to stress or mental health concerns, leading to an average loss of 2.4 workdays per employee [3]. Moreover, about 77% of employees reported that work stress had a negative impact on their physical health [4]. Therefore, among a wide range of mental health concerns, burnout has emerged as a critical driver of productivity loss. Its prevalence signals a deeper systemic issue, with costs extending beyond absenteeism to increased health care expenses, diminished employee engagement, and higher turnover rates [5-11]. In a recent meta-analysis study reviewing data from 5022 participants, nearly half of the participants reported elevated levels of burnout [12]. A survey conducted by the Canadian Medical Association on 4000 physicians and medical learners in 2021 showed that 53% of the participants indicated elevated levels of burnout during the pandemic. This number was 30% in 2017, pre-pandemic. Additionally, nearly half of the participants expressed intentions to reduce their clinical workload within the next 2 years [13]. Given the wide-ranging consequences, burnout should not be viewed solely as an individual health issue but as a strategic organizational challenge requiring timely and practical action to safeguard both employee well-being and overall productivity.

Despite growing awareness, a substantial gap remains between the demand for and access to mental health services. Even prior to the COVID-19 pandemic, mental health challenges were identified as a leading cause of loss of productivity and disability [14]. The World Health Organization has similarly announced that depression symptoms are the primary contributors to loss of productivity globally [15]. In the wake of the pandemic, the urgency of addressing mental health in the workplace was even more pronounced. Employers increasingly recognized the need to support psychological well-being by promoting mental health and providing resources for employees experiencing distress [16]. The prevalence of mental health issues has risen sharply since the onset of the COVID-19 pandemic, with anxiety rates increasing 4-fold (from 5% to 20%) and depression rates more than doubling (from 4% to 10%) [17]. Insurance data further reflect this trend, showing a 25% increase in claims for mental health support in 2020 [18]. Moreover, a recent survey revealed a 6% rise in the number and 12% in the duration of short-term mental health−related disability claims in 2021 [19]. Overall, mental health issues, including burnout, now account for approximately 30%-40% of short-term disability claims [20].

Burnout is defined as a multidimensional occupational phenomenon characterized by chronic work-related stress that has not been successfully managed [21]. It primarily encompasses 3 core dimensions: emotional exhaustion, cynicism, and reduced personal accomplishment [22]. Emotional exhaustion refers to feelings of being overextended and overstretched and depleted of one’s emotional resources, often resulting from excessive job demands and prolonged exposure to stressors [23]. Cynicism, or depersonalization, involves a detached or negative attitude toward one’s work and the people served, as it signifies an individual’s attempt to cope with overwhelming emotional demands by distancing themselves from their professional role [24]. The third dimension, reduced personal accomplishment, captures a decline in one’s sense of competence and achievement at work, often leading to a sense of inefficacy and a diminished capacity to perform job responsibilities effectively [25-28].

In addition to examining the alarming prevalence of workplace stress and burnout, it is equally important to evaluate the current solutions being used to address these challenges. Burnout interventions are generally classified into 2 main categories: organizational-level and employee-level support. Organizational-level support refers to the strategies and policies that companies implement that are aimed at fostering a work environment where employees can thrive and reduce exposure to excessive stress and burnout [29,30]. At its core, these initiatives are typically embedded in the company’s culture and leadership practices [31]. Employee-level support primarily consists of extended health benefits, including access to counseling and psychological therapies that are designed to improve the psychological health and well-being of individual employees. These resources are generally made available for employees and working adults to access at their own discretion. Numerous studies have demonstrated the effectiveness of these programs [32-34].

Despite the effectiveness and availability of these traditional evidence-based burnout interventions [35-38], several key obstacles impede employees’ access and engagement. First, financial constraints, both at the organizational level (budget caps on program funding) and the individual level (copays or out-of-pocket costs), can prevent participation or engagement with these programs [39]. Second, time demands present a significant hurdle: structured programs often require multisession commitments during standard business hours, creating scheduling conflicts or necessitating employees and working adults to sacrifice personal time for attendance [40]. Third, systemic wait lists for in-person services can delay support by weeks or months, undercutting the immediacy of the intervention when stress is most acute [41]. Finally, stigma surrounding mental health in the workplace, whether fear of being perceived as weak by colleagues or concerns about confidentiality, further depresses uptake, even when services are fully funded and readily available [39]. Collectively, these barriers contribute to persistently low participation rates and call into question the scalability of traditional support models.

To overcome these limitations and improve the scalability of mental health support programs, there has been growing interest in digital self-guided mental health programs [42]. Self-guided programs offer a flexible, accessible, and cost-effective complement to traditional burnout interventions. These programs empower working adults to engage with content at their own pace or convenience through an online platform that incorporates evidence-based techniques such as mindfulness, cognitive restructuring, and stress management [38]. By eliminating barriers like high cost, long wait times, scheduling conflicts, and the stigma sometimes associated with seeking professional help, self-guided approaches enable employees and working adults to take proactive control of their psychological wellness. Moreover, the self-paced nature of these interventions allows users to revisit material as needed and tailor practices to fit their unique circumstances.

Although prior reviews have provided valuable insights into the effectiveness of digital interventions targeting various psychological outcomes [43-48], they have largely overlooked the theoretical foundations, rationale, and specific content or modules that contribute to these positive effects. The present systematic review differs by focusing on the underlying theories, rationales, and intervention components that characterize effective self-guided digital interventions in the workplace and their effects on burnout, stress, and psychological well-being. While a recent systematic review and meta-analysis examined the mechanisms of change in digital interventions for clinical depression [49], that work reviewed 11 digital interventions targeting a clinical population. In contrast, the current review aims to identify the theoretical bases and content elements of self-guided digital interventions designed to prevent burnout and enhance psychological well-being among generally healthy working adults.

This systematic review is primarily focused on self-guided digital interventions since other intervention options, such as hybrid or guided digital interventions, still present several practical limitations that restrict their scalability and accessibility in workplace settings. Moreover, guided formats typically require ongoing facilitator involvement, which reintroduces cost, scheduling, and staffing challenges similar to those of traditional in-person interventions [50]. In contrast, fully self-guided programs remove the dependency on human facilitation, allowing organizations to reach a larger employee populations [51,52]. Furthermore, self-guided interventions provide a higher degree of privacy and autonomy, which may be particularly valuable in work cultures where mental health stigma remains prevalent [53]. Focusing on self-guided interventions thus allows this review to address an emerging yet underexplored domain in occupational health programs.

This systematic review aims to offer a comprehensive, transparent, and replicable synthesis of literature on the content and theoretical explanations of these digital self-guided mental health interventions in the workplace. Specifically, the review will focus on identifying the most effective content, activities, and assignments within these digital interventions that contribute to preventing burnout and improving psychological wellness. To ensure a rigorous and methodologically sound process, the review will follow the PRISMA (Preferred Reporting Items for Systematic Reviews and Meta-Analyses) guidelines [54,55].

### Review Aim

The aim of this systematic literature review is to identify the most effective content, design features, activities, and assignments included in brief digital self-guided mental health interventions for the workplace. Specifically, the review will assess which components are most effective in preventing burnout and improving psychological wellness, as well as their theoretical background and the rationale behind their effectiveness.

### Review Objectives

This review has 2 objectives: (1) to catalog and describe the content and components of each self-guided digital intervention: one of the key goals of this systematic review is to identify and detail the content, design features, activities, and assignments used in brief self-guided mental health interventions within workplace settings, focusing on those aimed at preventing burnout and enhancing psychological wellness. (2) To examine the theoretical underpinnings and rationale of each self-guided digital intervention: this systematic review will investigate the theoretical frameworks informing these digital interventions and determine how the underlying theory or mechanisms are associated with greater effectiveness in achieving mental health outcomes.

## Methods

### Study Design

This systematic review aims to offer a comprehensive, transparent, and replicable synthesis of the literature on the content and theoretical explanations of brief digital mental health interventions in the workplace. This systematic review protocol has been developed according to the PRISMA-P (Preferred Reporting Items for Systematic Reviews and Meta-Analyses for Protocols) checklist [56] (Checklist 1)(). This protocol has been registered on PROSPERO International Prospective Register of Systematic Reviews with the ID CRD420251035459.

### Search Methods

A comprehensive review of the literature will be conducted using 4 major relevant academic databases, including PsycINFO, PubMed, Web of Science, and Cochrane Library. These databases were selected to ensure a broad coverage of psychological research (PsycINFO), biomedical and clinical studies (PubMed), multidisciplinary citation indexing (Web of Science), and high-quality evidence syntheses (Cochrane Library). The search was conducted to identify records that match our inclusion criteria published between January 2000 and the date of commencement. This timeline was chosen as this review focuses on rapidly evolving digital mental health interventions. Given the fast-paced development of new technologies, studies prior to 2000 are unlikely to remain relevant to the aim of this systematic review. A systematic search will be conducted across multiple databases using a combination of search terms related to the target population (working adults), the mode of intervention delivery (digital, web-based, or mobile interventions), the intended outcomes (burnout prevention, stress reduction, and mental well-being), and the study design (randomized controlled trials and other experimental studies). The full list of search strings used for each database is provided in Table 1. Search terms will be applied to titles, abstracts, and keywords. Additionally, reference lists of included studies and relevant systematic reviews will be manually screened to identify any additional potentially eligible studies.

**Table 1.** Complete list of terms used as a search method of the systematic review.

Cluster	Search string
Population	adult* OR worker* OR employee* OR “working population” OR workplace* OR occupational OR job OR staff OR personnel OR “work* place” OR occupation*
Intervention	“digital intervention*” OR “web-based intervention*” OR “online intervention*” OR “internet-based intervention*” OR “e-health” OR “ehealth” OR “m-health” OR “mhealth” OR “smartphone app*” OR “mobile app*” OR “computer-based” OR “email-based”
Outcome	burnout OR “burn-out” OR “occupational stress” OR “work-related stress” OR “emotional exhaustion” OR cynicism OR “reduced efficacy” OR “stress” OR “work* stress” OR “exhaustion” OR “fatigue” OR “mental health” OR “depression” OR “anxiety” OR “wellness” OR “well-being” OR “wellbeing”
Design	“randomized controlled trial*” OR RCT OR “controlled clinical trial*” OR “quasi-experiment*” OR “experimental stud*” OR “control group”

### Screening and Selection Process

The search results from the selected databases will be imported into a reference management software (version 7; Zotero, developed by Digital Scholar) to facilitate duplicate detection and streamline the screening process. Papers will be managed using the web platform Rayyan [57], which is a web solution that expedites the initial screening of abstracts, followed by a 2-stage screening process. First, all duplicates will be removed. Second, the titles and abstracts will be reviewed for eligibility, and full texts will be retrieved for studies where eligibility remains unclear. Then, the full texts will be assessed based on the inclusion and exclusion criteria, considering factors such as intervention type, study population, and reported outcomes. Reasons for exclusion will be recorded at each stage of the selection process. As this study is part of a PhD student’s thesis, the initial search and eligibility assessment of retrieved articles will be conducted by the PhD student (EE). Any articles with uncertain eligibility will be discussed with my coauthor (JF). In instances where discrepancies or disagreements arise, RAH will be consulted to provide additional input and facilitate consensus.

### Eligibility Criteria

This systematic review will include studies that focus on digital, web-based health interventions aimed at addressing burnout or workplace wellness in working adults (aged 19 years and older) who are generally healthy and have no diagnosed clinical conditions, in order to evaluate preventive strategies and ensure that findings are broadly applicable to the wider workforce rather than being driven by treatment-seeking clinical samples. Eligible studies must examine workplace-related populations, including part-time and full-time employees. To ensure methodological rigor, only randomized controlled trials, experiments, or quasi-experiments with a control group (eg, no treatment, treatment as usual, waitlist control, or active control) will be included. Studies must assess intervention effectiveness using at least 1 pre- and postintervention measurement and report quantitative outcome data. The primary outcomes of this review include burnout, perceived stress, and psychological well-being, and the secondary outcomes include depression and anxiety symptoms. Interventions must be delivered through digital platforms such as websites, emails, computer programs, or smartphone apps, with active components included. Additionally, studies must describe the intervention’s content, modules, or activities in sufficient detail. Only peer-reviewed journal articles or dissertations published after 2000 will be considered to ensure studies included have undergone a rigorous editorial and methodological vetting process, thereby maximizing the quality and reliability of our findings [58]. Finally, both the abstract and full text must be available in English to allow for comprehensive review and analysis. The full list of eligibility and exclusion criteria is shown in Table 2.

**Table 2.** Inclusion and exclusion criteria of the systematic review.

Element	Eligibility criteria	Exclusion criteria
Population	Age: Adults (19 y old or over) Working population (part-time, full-time) and related to workplace Participants are generally healthy	Individuals who are unemployed, retired, or on disability leave Participants involved in young or senior care mental health contexts Individuals with psychiatric or complex mental health conditions (eg, PTSD^a^, schizophrenia, or comorbid substance misuse)
Intervention	Digital or web-based health interventions, where active elements are delivered via website, email, computer program, or smartphone app Intervention content or modules or activities are described	Interventions using tracking devices or wearable technologies (eg, biofeedback) Interventions incorporating virtual reality or augmented reality Telehealth or videoconferencing interventions where technology is only a mode of communication (eg, Skype, e-counseling) Text or SMS-based health interventions Interventions where homework is digital, but the main intervention is delivered in-person
Comparison	Studies must include a control group (eg, nontreatment, treatment-as-usual, waitlist control, or active control)	Studies without a control group
Outcome	· Burnout measured as an outcome (directly or indirectly) Reported quantitative effectiveness outcomes Included at least one pre- and 1 post-intervention assessment	Studies focusing on risk factors, protective factors, motivation, or willingness to participate Studies focused on participant satisfaction, sources of stress, or burnout prevention strategies Studies assessing organizational- or system-level strategies or initiatives only Protocol papers or cost-analysis studies
Study design	Randomized controlled trials (RCTs), experiments, or quasi-experimental designs Minimum of 5 participants	Observational, qualitative, or correlational studies Case studies with fewer than 5 participants
Publication type	Published in peer-reviewed journals or dissertations Published post-2000 Abstract and full text available in English	Gray literature, opinion pieces, protocols, and reviews

a PTSD: posttraumatic stress disorder.

### Data Extraction Process

For each eligible study, we will extract key data into a structured Microsoft Excel template to ensure consistency and accuracy [59]. This will include (1) Authors and References: Names of the authors and publication year; (2) Sample: Sample size, target population (eg, employees, industry type), and setting (eg, online, workplace); (3) Methodology or Study Design: Research design used in the study (eg, randomized controlled trial, quasi-experimental), including details about the control group (active control, passive waitlist group, or treatment as usual); (4) Objective or Goal: The primary aim of the intervention, such as reducing burnout or improving psychological wellness; (5) Outcomes or Measures: Instruments used to assess effectiveness; (6) Type of Intervention: Focus and delivery method (eg, digital self-guided mental health program, web-based training, mobile application); (7) Theoretical Framework: The underlying model or framework guiding the intervention (eg, Cognitive Behavioral Therapy, Mindfulness-Based Stress Reduction); (8) Content: A brief description of the intervention’s structure, components, and activities; (9) Effectiveness: A brief summary of the quantitative results indicating its effectiveness and effect sizes; and (10) Fidelity: A brief summary of whether the intervention was implemented as intended and adhered to the planned structure. This systematic extraction process will ensure a comprehensive and standardized analysis of all relevant aspects of the interventions.

### Risk of Bias Assessment

The risk of bias (ROB) will be assessed using the revised Cochrane Risk-of-Bias (RoB 2; version 2) tool for randomized trials [60]. Each study will be evaluated across the 5 RoB 2 domains: (1) bias resulting from the randomization process, (2) bias resulting from deviations from intended interventions, (3) bias due to missing outcome data, (4) bias in outcome measurement, and (5) bias in the selection of reported results. The primary author will perform the initial ROB assessment. Included studies will be classified as having “low risk,” “some risk,” “high risk,” or “unclear risk.”

### Data Analysis

An integrated narrative synthesis will be conducted to investigate and explore the connection between the findings within and across the included interventions as recommended by Popay et al [61]. This structured iterative approach consists of 6 key steps: (1) developing an overarching theoretical understanding, (2) conducting an initial analysis, (3) examining relationships between findings, (4) evaluating the strength and consistency of the evidence, and (5) drawing conclusions and (6) practical recommendations.

To facilitate this synthesis, various analytical techniques will be employed, including content analysis, tabulation, concept mapping, and critical reflection. The preliminary synthesis will involve summarizing study findings and categorizing them into meaningful themes through content analysis and tabulation. Relationships within and across studies will then be explored using concept mapping and visual representations to enhance conceptual and methodological integration. Finally, a critical reflection and quality appraisal process will be conducted. The GRADE (Grading of Recommendations Assessment, Development, and Evaluation) framework will be used to evaluate the quality of evidence within each study and its recommendations [62].

More specifically, objective 1 (to catalog and describe the content and components of interventions) will be primarily analyzed through content analysis and tabulation by including the characteristics of selected studies, such as intervention goals, delivery format, duration, activities, and behavioral assignments. These entries will then be grouped into broader categories that capture recurring design features and intervention components. Additionally, objective 2 (to examine the theoretical underpinnings and rationale of each intervention) will be analyzed by linking each intervention’s theoretical framework or psychological mechanism (eg, cognitive-behavioral theory, mindfulness, self-determination theory) to its reported outcomes. Quantitative findings, such as effect sizes and direction of effects, will be compared across studies sharing similar theoretical foundations and levels of evidence quality (including ROB) to determine which frameworks and mechanisms are most strongly associated with significant improvements in burnout and psychological wellness outcomes. Finally, a critical reflection will be applied to interpret how contextual factors (such as setting, population, and intervention format) may influence the effectiveness of each theoretical approach.

The synthesis of the findings will lead to evidence-based recommendations on the content and theories behind digital interventions for psychological wellness in the workplace.

## Results

In April 2025, the study protocol was submitted to PROSPERO. Shortly after, we conducted preliminary searches on the academic databases. As of October 2025, the title and abstract screening and full-text screening have been completed, and data analysis and reporting have commenced. This systematic review is anticipated to be completed by May 2026.

## Discussion

### Principal Findings

This systematic review is positioned to make a meaningful contribution to the field of digital mental health interventions in workplace contexts. This study will identify the “active ingredients” and underlying theoretical mechanisms that underpin the effectiveness of self-guided digital interventions that can be applied in organizational settings.

Although the results are pending, this proposed systematic review is expected to provide the most comprehensive analysis of how self-guided digital mental health interventions function for employees and working adults. We also anticipate identifying substantial growth in publications, particularly in the past decade, which signals the field’s rapid maturation and growing recognition of digital interventions as a legitimate therapeutic modality to improve employee mental health outcomes beyond experimental approaches [63].

The review is expected to reveal core intervention components that characterize effective programs, including psychoeducational modules, skill-building exercises, multimedia integration, and personalization features. We anticipate that effective interventions will demonstrate sophisticated adaptation of evidence-based psychological frameworks, such as cognitive behavioral therapy, acceptance and commitment therapy, social cognitive theory, and positive psychology, to digital formats while maintaining theoretical fidelity. Additionally, the review is also expected to uncover and capture implementation challenges such as engagement barriers, time constraints, privacy concerns, and variations in organizational support that may influence intervention effectiveness [64]. By focusing exclusively on self-guided formats, this review will shift attention from resource-intensive guided interventions to scalable solutions that could overcome traditional barriers to mental health support in organizational settings [65].

### Comparison to Previous Work

Existing literature on workplace mental health interventions has established the efficacy of various psychological frameworks, including cognitive behavioral therapy and mindfulness-based approaches, in reducing occupational stress and burnout [66-68]. However, the translation of these frameworks to fully self-guided digital formats remains understudied. Our review builds upon foundational work in digital mental health while extending the focus to implementation characteristics, theoretical mechanisms, and contextual factors unique to self-guided workplace interventions. This targeted approach is expected to reveal insights into the minimum viable components necessary for effective self-guided digital interventions, the role of personalization and adaptation, and the organizational conditions that facilitate or hinder intervention success.

### Strengths, Limitations, and Future Directions

This systematic review protocol possesses several methodological strengths that will enhance the reliability and comprehensiveness of the findings. The multidatabase search strategy (PubMed, Web of Science, PsycINFO, Cochrane) combined with additional source identification through existing systematic reviews will minimize publication bias and ensure broad literature coverage. The temporal scope spanning from 2000 will capture the evolution of digital mental health technologies and their application in workplace contexts.

The review’s exclusive focus on self-guided interventions addresses a critical research gap, as these formats offer unique scalability advantages essential for widespread implementation in organizational settings. The planned detailed analysis of intervention components, delivery mechanisms, and theoretical foundations will provide valuable insights for both researchers and practitioners. Additionally, the comprehensive data extraction protocol will enable a nuanced understanding of how various design features and implementation contexts influence intervention effectiveness.

However, several anticipated limitations warrant consideration. The exclusion of guided or blended interventions, while maintaining methodological focus, may overlook important insights about the continuum of support needed in digital workplace mental health programs. Self-guided interventions may face unique challenges, including lower engagement rates compared to guided formats, and the review may reveal limited information about implementation contexts, organizational support structures, and workplace-specific adaptations. The predominance of studies from high-income countries may also limit the generalizability of the findings to diverse global workplace contexts.

The anticipated contribution of this review is multifaceted, advancing both academic understanding and practical application of self-guided digital mental health interventions in workplace settings. For the research community, this review will provide a comprehensive synthesis of the current evidence base, identify critical knowledge gaps, and establish a foundation for future comparative effectiveness studies. The detailed analysis of theoretical frameworks and intervention components is expected to inform the development of next-generation digital interventions that leverage emerging technologies while maintaining evidence-based foundations.

For organizational stakeholders and practitioners, this review will offer practical guidance on selecting, implementing, and evaluating self-guided digital mental health programs, enabling evidence-informed decision-making about digital health investments through the identification of core intervention components and implementation facilitators. The findings will be particularly valuable for small- and medium-sized enterprises that may lack resources for traditional employee assistance programs yet seek scalable, accessible mental health solutions. From a policy perspective, the review will provide essential evidence for developing standards and guidelines that ensure workplace digital mental health programs balance innovation with ethical safeguards and appropriate oversight. By synthesizing effectiveness data, implementation considerations, and quality indicators, the review will inform policy frameworks that promote equitable access to evidence-based digital interventions across diverse workplace populations. Future research directions will likely include investigations into optimal intervention dosage and duration, comparative analyses of theoretical frameworks across similar populations, and evaluations of implementation strategies that enhance engagement and effectiveness. Moreover, the anticipated findings will underscore the need for standardized outcome measures and reporting protocols to facilitate meta-analytic synthesis, while recognizing that emerging technologies—such as artificial intelligence and virtual reality—require ongoing, rigorous evaluation to ensure that innovation remains evidence-based and continues to expand scalable, effective workplace mental health support.

### Conclusion

The findings of this systematic review will inform the design of innovative, scalable, and accessible interventions aimed at enhancing psychological well-being and reducing burnout among working adults.

The significance of this review extends beyond documenting intervention effectiveness to addressing fundamental questions about the role of self-guided digital support within comprehensive workplace mental health strategies. As the global workforce continues to face unprecedented stressors and organizations seek scalable solutions to support employee well-being, understanding the capabilities and limitations of self-guided interventions becomes increasingly critical. The review’s findings are expected to challenge traditional assumptions about the necessity of human guidance in digital mental health interventions while identifying the conditions under which self-guided support can achieve meaningful clinical and organizational outcomes.

Ultimately, this systematic review will contribute to the transformation of workplace mental health support from a reactive, resource-intensive model to a proactive, accessible, and scalable approach that can reach diverse employee populations. This proposed review has the potential to advance our understanding of how digital tools can create psychologically healthy workplaces that not only prevent burnout and distress but also actively promote employee thriving and organizational success. The knowledge generated through this systematic review will be instrumental in realizing the potential of digital mental health interventions to address the growing mental health crisis in workplace settings worldwide.

## Supplementary material

10.2196/80417 Checklist 1 PRISMA-P checklist.

## Abbreviations:

GRADE: Grading of Recommendations Assessment, Development and Evaluation
PRISMA: Preferred Reporting Items for Systematic Reviews and Meta-Analyses
PRISMA-P: Preferred Reporting Items for Systematic Reviews and Meta-Analyses for Protocols
ROB: risk of bias
